# Association of neighborhood-level socioeconomic disadvantage and Life's Essential 8 in early pregnancy

**DOI:** 10.1016/j.ajpc.2024.100925

**Published:** 2024-12-24

**Authors:** Kartik K. Venkatesh, William A. Grobman, Xiaoning Huang, Lynn M. Yee, Janet Catov, Hy Simhan, David M. Haas, Brian Mercer, Uma Reddy, Robert M. Silver, Lisa D. Levine, Judith Chung, George Saade, Philip Greenland, C. Noel Bairey Merz, Becky McNeil, Sadiya S Khan

**Affiliations:** a Department of Obstetrics and Gynecology, The Ohio State University, Columbus, OH, USA; b Department of Preventive Medicine, Northwestern University, Chicago, IL, USA; c Department of Obstetrics and Gynecology, Northwestern University, Chicago, IL, USA; d Department of Obstetrics and Gynecology, University of Pittsburgh, Pittsburgh, PA, USA; e Department of Obstetrics and Gynecology, Indiana University, Indianapolis, IN, USA; f Department of Obstetrics and Gynecology, Case Western Reserve University, Cleveland, OH, USA; g Department of Obstetrics and Gynecology, Columbia University, New York, NY, USA; h Department of Obstetrics and Gynecology, University of Utah, Salt Lake City, UT, USA; i Department of Obstetrics and Gynecology, University of Pennsylvania, Philadelphia, PA, USA; j Department of Obstetrics and Gynecology, University of California, Irvine, Orange, CA, USA; k RTI International, Durham, NC, USA; l Department of Obstetrics and Gynecology, Eastern Virginia Medical College, Norfolk, VA, USA; m Barbra Streisand Women's Heart Center, Smidt Heart Institute, Cedars Sinai Medical Center, Los Angeles, CA, USA

**Keywords:** Pregnancy, Cardiovascular health, Life's Essential 8, Area deprivation index, Social determinants of health

## Abstract

We examined whether neighborhood-level socioeconomic disadvantage per the Area Deprivation Index (ADI) was associated with maternal cardiovascular health (CVH) in early pregnancy per the American Heart Association Life's Essential 8 (LE8). This is a cross-sectional analysis from the prospective Nulliparous Pregnancy Outcomes Study-Monitoring Mothers-to-Be Heart Health Study (nuMoM2b-HHS) cohort. The exposure was the ADI in tertiles (T) from least (T1) to most (T3) socioeconomic disadvantage. The outcome was the LE8 as a continuous score ranging from worst (0) to best (100) composite CVH; and included physical activity, diet quality, tobacco use, sleep quantity, body mass index, blood pressure, glucose, and lipid levels. Among 4,508 nulliparous individuals at a mean maternal age of 27.0 years (SD: 5.6) and at a mean gestational age of 11.4 weeks (SD 1.6), the mean ADI was 48.0 (SD: 30.4) and the mean LE8 was 80.3 (SD: 12.5). Pregnant individuals living in neighborhoods with greater socioeconomic disadvantage had lower mean LE8 scores (i.e., worse CVH) compared with those living in neighborhoods with lesser disadvantage (T1 vs. T2 adjusted mean: 82.6 vs. 80.5; adj. ß:2.08; 95 % CI:3.51, -0.64; and T1 vs. T3 adjusted mean: 82.6 vs. 77.8; adj. ß:4.77; 95 % CI:8.16, -1.38). Neighborhood-level socioeconomic disadvantage was associated with worse maternal CVH in early pregnancy.

## Highlights

• The relationship between neighborhood-level social determinants of health and Life's Essential 8 remains to be examined in pregnancy.
• In a multisite US cohort of individuals in their first pregnancy, neighborhood-level socioeconomic disadvantage per the Area Deprivation Index was associated with worse maternal cardiovascular health per the Life's Essential 8 in early pregnancy.
• Future studies should explore whether assessment of neighborhood-level social determinants of health and cardiovascular health could be integrated into pregnancy care to improve outcomes.

## 1 Introduction

Poor cardiovascular health (CVH) is a leading contributor to adverse pregnancy outcomes and severe maternal morbidity and mortality. [1] Nearly 70 % of pregnant individuals in the United States (US) enter pregnancy with suboptimal CVH, [2] which occurs more frequently among those who experience a higher burden of adverse social determinants of health. [3,4] The American Heart Association (AHA) proposed the Life's Essential 8 (LE8) framework in 2022 to promote CVH to reduce the risk of developing cardiovascular disease. [3] The LE8 includes eight metrics, including four health behaviors (smoking, physical activity, sleep, and diet) and four health factors (cholesterol, blood pressure, glucose, and body mass index. [BMI]).

Social determinants of health (SDOH) can be assessed at an individual-, interpersonal-, or neighborhood-level. [5,6] The Area Deprivation Index (ADI) is a composite measure of neighborhood-level socioeconomic disadvantage, [7] and a higher ADI (i.e., worse disadvantage) is associated with an increased risk of adverse pregnancy outcomes and poor CVH outside of pregnancy. [[8], [9], [10]] The relationship between the ADI and LE8 has not been examined in pregnancy. [11] Understanding this association provides an opportunity for equitable CVH prevention and intervention as part of prenatal care. [6]

The objective of the current analysis was to examine whether greater neighborhood socioeconomic disadvantage per the ADI was associated with worse maternal CVH per the LE8 in early pregnancy.

## 2 Methods

This is a secondary analysis from the prospective Nulliparous Pregnancy Outcomes Study-Monitoring Mothers-to-Be Heart Health Study (nuMoM2b-HHS) cohort. [8,12] Individuals were enrolled in the first trimester at eight U.S. medical centers (Case Western Reserve University, Columbia University, Indiana University, University of Pittsburgh, Northwestern University, University of California at Irvine, University of Pennsylvania, and the University of Utah) from October 2010 to September 2013 (ClinicalTrials.gov NCT01322529). Data were centrally managed by the Data Coordinating and Analysis Center at RTI International. Key inclusion criteria included no prior history of delivery at 20 weeks’ gestation or later, viable singleton gestation between 6 + 0 to 13 + 6 weeks, and no major genetic or structural anomalies. Each site's institutional review board approved the study before initiation and all participants gave written, informed consent.

The current cross-sectional analysis used data from the first visit (completed 6 to 13 weeks’ gestation) during which study personnel administered structured questionnaires to ascertain data on demographic characteristics, medical history, dietary history, and psychosocial factors. Trained personnel also abstracted data from the clinical chart. Each participant's residential address was collected and geocoded.

The exposure was ADI (https://www.neighborhoodatlas.medicine.wisc.edu/), which was converted to a rank based on a locale's national percentile. [7] Home addresses in the first trimester were geocoded at the census-tract level and linked to the 2015 ADI. The ADI was scored from 0 (low deprivation) to 100 (high deprivation) and standardized to the US population. The ADI was analyzed in tertiles from the lowest ADI (i.e., least deprivation [tertile T1, reference]) to the highest ADI (i.e., most deprivation [tertile 3, T3]).

The primary outcome was overall CVH defined by the LE8 in the first trimester. The LE8 was assessed by self-report for physical activity (minutes per week), diet quality (standardized Healthy Eating Index-2010 [HEI-2010]), tobacco use (current use), sleep quantity (hours per week); and was measured for BMI (kg/m^2^), systolic and diastolic blood pressure (mm Hg), glucose (mg/dL), and non-HDL cholesterol (non-fasting, mg/dL), each reported as a mean unless otherwise noted (Appendix Table 1). Each of the 8 components was given a score, with the cumulative LE8 score ranging from 0 (worst) to 100 (best). [3] Secondarily, each CVH component was assessed separately.

Multivariable linear regression was used and adjusted for individual-level covariates informed by recent causal frameworks of neighborhood-level SDOH and CVH, [11] including: maternal age (continuous), self-reported race and ethnicity as a SDOH reflecting racism (non-Hispanic White, non-Hispanic Black, Hispanic, non-Hispanic Asian, and other), and highest level of educational attainment (high school or less; college inclusive of some college credit, but no degree; college graduate; and greater than college inclusive of a graduate degree). We used multiple imputation by chained equations using the STATA “mi impute chained” package for missing data. [13,14] We created 10 imputed datasets. For the LE8 outcome, imputation varied between 1.7 % for BMI to 31.0 % for physical activity (Appendix Table 2). A sensitivity analysis was performed that included only those cases with complete primary exposure and outcome ascertainment (N = 2134).

## 3 Results

This analysis included the 4508 individuals enrolled in the nuMoM2b-HHS cohort. The mean age was 27.0 years (standard deviation [SD]: 5.6), 50.4 % had a college education, 54.6 % had private health insurance, and 13.8 % self-identified as non-Hispanic Black and 16.3 % as Hispanic (Appendix Table 3). The mean gestational age at assessment was 11.4 weeks (SD: 1.6).

The mean ADI was 48.0 (SD: 30.4). Age, educational attainment, insurance coverage, and racial and ethnic identity varied by ADI tertile (chi square *p* < 0.001 for all) (Appendix Table 4).

The adjusted mean LE8 score was 80.3 (95 % CI: 78.8, 81.8) and the mean scores for the individual eight components are presented in Table 1 (Appendix Table 3).

**Table 1:** Associations between ADI tertiles and overall and component Life's Essential 8 (LE8) scores in early pregnancy.

	Diet	Physical activity	Tobacco use	Sleep	BMI	Non-HDL cholesterol	Glucose	Blood pressure	Overall LE8
Classification (points)[2]	Percentile score on the HEI-2010	Minutes per week	Smoking status	Hours per night	kg/m^2^	mg/dL	mg/dL	Systolic/diastolic, mmHg	%
Unadjusted Least Square Means (95 % CI)^1^									
Overall	70.36, (66.20, 74.52)	71.39, (67.58, 75.20)	72.42, (63.11, 81.73)	88.43, (87.24, 89.62)	73.17, (70.22, 76.13)	86.87, (84.77, 88.98)	89.73, (88.22, 91.25)	90.11, (87.92, 92.30)	80.31, (78.46, 82.16)
ADI Tertile 1, (low deprivation)	79.80, (75.36, 84.24)	76.96, (69.91, 84.01)	76.53, (67.16, 85.90)	90.88, (88.88, 92.88)	81.63, (77.20, 86.06)	85.90, (83.65, 88.14)	91.07, (89.13, 93.01)	91.88, (88.93, 94.82)	84.33, (82.53, 86.13)
ADI Tertile 2	72.54, (68.04, 77.03)	71.31, 66.75, 75.87)	73.64, (62.52, 84.76)	88.90, (87.00, 90.80)	73.46, (69.89, 77.02)	86.33, (83.54, 89.12)	89.84, (86.84, 92.84)	89.41, (87.49, 91.33)	80.68, (78.49, 82.87)
ADI Tertile 3, (high deprivation)	58.42, (49.34, 67.50)	65.68, (61.02, 70.34)	66.94, (52.16, 81.73)	85.43, (83.56, 87.31)	64.11, (58.08, 70.14)	88.41, (85.54, 91.28)	88.24, (86.15, 90.33)	88.96, (84.64, 93.27)	75.77, (71.58, 79.97)
Least Square Means, (95 % CI)[1]									
Overall	70.37, (66.82,73.92)	71.40, (68.17,74.62)	72.42, (63.46,81.37)	88.44, (87.70,89.17)	73.17, (71.16,75.19)	86.88, (85.09,88.66)	89.63, (87.72,91.55)	90.11, (88.00,92.22)	80.30, (78.78,81.83)
ADI Tertile 1, (low deprivation)	73.89, (71.51, 76.27)	73.31, (68.55, 78.06)	74.96 (66.16,83.75)	89.26, (87.29, 91.23)	79.26, (75.66, 82.86)	86.96, (84.46, 89.46)	91.33, (89.01, 93.64)	91.49, (89.06, 93.93)	82.56, (81.28, 83.84)
ADI Tertile 2	71.69, (66.84, 76.54)	70.81, (66.16, 75.46)	74.16 (64.78,83.54)	88.46, (86.63, 90.29)	72.65, (70.64, 74.66)	86.94, (84.42, 89.46)	89.66, (86.40, 92.93)	89.47, (87.23, 91.71)	80.48, (78.54, 82.42)
ADI Tertile 3, (high deprivation)	65.43, (60.11, 70.74)	69.98, (65.44, 74.53)	68.07 (56.02,80.11)	87.55, (85.64, 89.47)	67.37, (62.00, 72.74)	86.72, (83.90, 89.55)	87.84, (84.73, 90.95)	89.30, (86.30, 92.30)	77.78, (74.61, 80.96)
Unadjusted Beta coefficient (95 % CI)									
ADI Tertile 1, (low deprivation)	Ref.	Ref.	Ref.	Ref.	Ref.	Ref.	Ref.	Ref.	Ref.
ADI Tertile 2	−7.26*, (−12.18, −2.34)	−5.65, (−12.18, 0.88)	−2.89, (−9.58, 3.80)	−1.98, (−4.50, 0.55)	−8.18*, (−12.72, −3.64)	0.43, (−2.90, 3.77)	−1.23, (−4.44, 1.98)	−2.46*, (−4.50, −0.43)	−3.65*, (−5.54, −1.77)
ADI Tertile 3, (high deprivation)	−21.38*, (−31.86, −10.90)	−11.28*, (−19.55, −3.01)	−9.59, (−24.84, 5.67)	−5.44*, (−8.05, −2.84)	−17.52*, (−25.97, −9.08)	2.51, (−0.63, 5.65)	−2.83, (−5.74, 0.08)	−2.92, (−8.20, 2.36)	−8.56*, (−13.30, −3.81)
Adjusted Beta coefficient, (95 % CI)[2]									
ADI Tertile 1, (low deprivation)	Ref.	Ref.	Ref.	Ref.	Ref.	Ref.	Ref.	Ref.	Ref.
ADI Tertile 2	−2.20, (−5.79, 1.39)	−2.49, (−8.24, 3.25)	−0.79, (−5.20, 3.62)	−0.80, (−3.50, 1.90)	−6.61*, (−9.81, −3.41)	−0.02, (−3.42, 3.38)	−1.67, (−4.81, 1.48)	−2.02*, (−3.64, −0.40)	−2.08*, (−3.51, −0.64)
ADI Tertile 3, (high deprivation)	−8.46*, (−12.95, −3.97)	−3.32, (−9.12, 2.48)	−6.89, (−16.83, 3.06)	−1.71, (−5.04, 1.62)	−11.89*, (−19.51, −4.27)	−0.24, (−4.14, 3.67)	−3.49, (−7.52, 0.54)	−2.20, (−5.40, 1.01)	−4.77*, (−8.16, −1.38)

1 Least Square Means and beta coefficients were based on ordinary least square regression models adjusted for maternal age (continuous), self-reported race and ethnicity as a social determinant (White, Black, Hispanic, Asian, other), and educational attainment (high school or less, some college or associate/tech, college or above), with imputation for missing covariates.

2 Appendix Table 1 provides the scoring for each of the eight LE8 measures.

⁎ *p* < 0.05. Standard errors are clustered on study sites. Abbreviations: ADI, area deprivation index; CI, confidence interval; BMI (body mass index). N = 4508.

Individuals living in neighborhoods with greater socioeconomic disadvantage had significantly lower mean LE8 scores (i.e., worse CVH) compared with those living in neighborhoods with lesser disadvantage (T1 vs. T2 adjusted mean: 82.6 vs. 80.5; adj. ß: −2.08; 95 % CI: −3.51, −0.64; and T1 vs. T3 adjusted mean: 82.6 vs. 77.8; adj. ß: −4.77; 95 % CI: −8.16, −1.38) (Table 1).

In secondary analyses, those living in neighborhoods with the greatest socioeconomic disadvantage (i.e., T3) had poorer diet quality and higher BMI; and those with an intermediate degree of neighborhood disadvantage (i.e., T2) had higher BMI and higher systolic and diastolic blood pressure compared with those living in neighborhoods with the least disadvantage (i.e., T1) (Table 1).

These results were similar in sensitivity analysis that did not use covariate data imputation (Appendix Table 5).

In secondary analyses, for the exception of the association between ADI and LE8 for Asian vs. White individuals (*p* = 0.01), there were no significant differences between each minoritized race and ethnicity group versus White individuals. This finding suggests that the greater frequency of being in neighborhoods with structural disadvantage among minoritized populations contributes to racial and ethnic differences.

## 4 Discussion

In a multi-center cohort of nulliparous pregnant individuals in the US, greater neighborhood-level socioeconomic disadvantage was associated with worse CVH as reflected in a lower LE8 score in early pregnancy. And by individual LE8 components, greater neighborhood socioeconomic disadvantage was associated with higher BMI, higher blood pressure, and poorer dietary quality.

The AHA LE8 is increasingly used as a preventative framework to assess and act on health behaviors and factors before adverse cardiovascular disease manifests [3]. Our findings using a composite and contemporary CVH metric extend what was previously known about associations between SDOH and individual CVH risk factors among pregnant individuals. [2,15] In pregnancy, the ADI has been associated with risk enhancing adverse pregnancy outcomes, including hypertensive disorders of pregnancy, [16] gestational diabetes mellitus, [17] abnormal infant birthweight, [8] and stillbirth. [18] Prior studies have assessed associations between individual- and neighborhood- SDOH have focused on the predicted risk of cardiovascular disease, or actual cardiovascular disease events decades after pregnancy. [4,5] Pregnancy is an important opportunity that should be prioritized to assess and optimize CVH. [19,20]

Recent professional society recommendations recognize the public health importance of CVH measurement, modification, and monitoring in pregnancy to improve outcomes across the life course for both the pregnant individual and exposed child. [19,20] Measures of CVH included in the LE8 are routinely assessed during prenatal care with the exception of cholesterol, and they are increasingly employed to frame CVH in pregnancy. [1] Therefore, the implementation of LE8 is well-primed for use in pregnancy. In addition, recently developed prediction models for cardiovascular disease integrate measures of place-based SDOH. [21]

A strength of this study is the assessment of multiple factors integrated in the LE8 summative score. The ADI is increasingly being used in CVH research and clinical practice among non-pregnant adults for assessment of a neighborhood's overall environment for SDOH. Both the LE8 and ADI can be measured with data in health records and can be used across healthcare settings.

Limitations of the study include that it is a cross-sectional analysis. Glucose levels were not obtained fasting, and hence may not fully reflect dysglycemia. LE8 was assessed only in early pregnancy in this cohort, and these results should also be examined later in pregnancy as well as postpartum. This cohort was restricted to individuals who enrolled in a longitudinal cohort early in a pregnancy in which they gave birth to their first child and were receiving care at larger US medical centers, and hence the results may not be generalize to all subgroups or settings.

Future studies should explore whether assessment of the ADI and LE8 could be integrated into pregnancy care pathways to address, and intervene to improve CVH among pregnant individuals who experience place-based social disparities. Whether individuals with a higher ADI in the peripartum period have improved CVH outcomes through structural interventions or enhanced care pathways requires further study.

## Author contributions

KV, WG, and SK wrote the manuscript. KV, WG, SK, and LY conceptualized the study question. KV, XH, BM, and SK conducted all statistical analyses. WG, DH, JC, HS, DH, BM, UR, RS, LD, JC, GS, PG, and NM led the study, collected the data, and contributed to designing the study and interpreting the data.

## CRediT authorship contribution statement

**Kartik K. Venkatesh:** Writing – review & editing, Writing – original draft, Validation, Supervision, Methodology, Investigation, Formal analysis, Data curation. **William A. Grobman:** Supervision, Investigation, Funding acquisition, Formal analysis, Conceptualization. **Xiaoning Huang:** Visualization, Software, Resources, Methodology, Formal analysis, Data curation. **Lynn M. Yee:** Writing – review & editing, Writing – original draft, Supervision, Methodology, Formal analysis. **Janet Catov:** Writing – review & editing, Methodology, Investigation, Conceptualization. **Hy Simhan:** Writing – review & editing, Project administration, Methodology, Investigation. **David M. Haas:** Writing – review & editing, Methodology, Investigation, Funding acquisition, Data curation. **Brian Mercer:** Supervision, Project administration, Investigation, Funding acquisition. **Uma Reddy:** Supervision, Project administration, Investigation, Funding acquisition, Data curation, Conceptualization. **Robert M. Silver:** Writing – review & editing, Project administration, Methodology, Investigation, Funding acquisition, Data curation. **Lisa D. Levine:** Writing – review & editing, Supervision, Project administration, Investigation, Data curation. **Judith Chung:** Supervision, Project administration, Investigation, Formal analysis, Data curation. **George Saade:** Supervision, Project administration, Investigation, Funding acquisition, Conceptualization. **Philip Greenland:** Writing – review & editing, Methodology, Investigation, Conceptualization. **C. Noel Bairey Merz:** Writing – review & editing, Methodology, Formal analysis, Conceptualization. **Becky McNeil:** Visualization, Validation, Project administration, Methodology, Funding acquisition, Formal analysis, Data curation, Conceptualization. **Sadiya S Khan:** Writing – review & editing, Writing – original draft, Validation, Supervision, Project administration, Methodology, Investigation, Formal analysis, Data curation, Conceptualization.

## Declaration of competing interest

The authors declare that they have no known competing financial interests or personal relationships that could have appeared to influence the work reported in this paper.

## Appendix Supplementary materials

Image, application 1
