# Supplementary material for: Association of neighborhood-level socioeconomic disadvantage and Life's Essential 8 in early pregnancy
Source: Am J Prev Cardiol. 2024 Dec 24;21:100925. doi: 10.1016/j.ajpc.2024.100925 (PMC11750432; doi:10.1016/j.ajpc.2024.100925)
Supplement: Supplementary file 1 [file mmc1.docx]

**APPENDIX**

| **Appendix Table 1. Cardiovascular health score based on 8 metrics of the composite American Heart Association Life’s Essential 8 (LE8)** | | |
| --- | --- | --- |
| **Cardiovascular Health Metric** | **Points** | **Level** |
| **Blood Pressure, systolic blood pressure / diastolic blood pressure (mmHg)** |  |  |
|  | 100 | <120 / <80 |
|  | 75 | 120-129 / <80 |
|  | 50 | 130-139 or 80-89 |
|  | 25 | 140-159 or 90-99 |
|  | 0 | >160 / >100 |
|  |  | *Subtract 20 points if treated level |
| **Body Mass Index (kg/m^2^)** |  |  |
|  | 100 | <25.0 |
|  | 70 | 25.0 to 29.9 |
|  | 30 | 30.0 to 34.9 |
|  | 15 | 35.0 to 39.9 |
|  | 0 | >40.0 |
| **Diet (Healthy Eating Index 2010 score)** |  |  |
|  | 100 | >95^th^ percentile |
|  | 80 | 75^th^ to 94^th^ percentile |
|  | 50 | 50^th^ to 74^th^ percentile |
|  | 25 | 25^th^ to 49^th^ percentile |
|  | 0 | 1^st^ to 24^th^ percentile |
| **Glucose (mg/dL)** |  |  |
|  | 100 | <100 |
|  | 60 | 100 to <126 |
|  | 0 | >126 or receiving treatment for and/or report diabetes |
| Nicotine exposure |  |  |
|  | 100 | Never smoker |
|  | 50 | Former smoker |
|  | 0 | Current smoker |
| Non-HDL Cholesterol Level (mg/dL) |  |  |
|  | 100 | <130 |
|  | 60 | 130 to 159 |
|  | 40 | 160 to 189 |
|  | 20 | 190 to 219 |
|  | 0 | >220 |
|  |  | *If drug-treated level, subtract 20 points |
| Physical Activity (minutes per week) |  |  |
|  | 100 | >150 |
|  | 90 | 120 to 149 |
|  | 80 | 90 to 119 |
|  | 60 | 60 to 89 |
|  | 40 | 30 to 59 |
|  | 20 | 1 to 29 |
|  | 0 | 0 |
| Sleep Health (hours per night) |  |  |
|  | 100 | 7 to <9 |
|  | 90 | 9 to <10 |
|  | 70 | 6 to <7 |
|  | 40 | 5 to <6 or >10 |
|  | 20 | 4 to <5 |
|  | 0 | <4 |

| **Appendix Table 2. Missing data and imputation^1^** | |
| --- | --- |
| **Variable** | **% Missing** |
| **Body mass index (kg/m2)** | 1.7 |
| **Systolic blood pressure (mmHg)** | 2.3 |
| **Diastolic blood pressure (mmHg)** | 2.3 |
| **Glucose (mg/dL)** | 2.6 |
| **Total cholesterol (mg/dL)** | 2.6 |
| **Non-HDL cholesterol (mg/dL)** | 2.6 |
| **Sleep duration (hours per night)** | 21.4 |
| **Healthy Eating Index (HEI)-2010** | 14.8 |
| **Physical activity (minutes per week)** | 31.0 |
| **Area deprivation index 2015** | 4.4 |
| ^1^Multivariate Imputation by chained equations were performed using linear regression models. After imputation, minimum body mass index was set to 15 kg/m^2^. Maximum physical activity was set to 600 min/week. Maximum ADI was set to 100. Negative HEI, physical activity, and ADI were converted to 0. | |

| **Appendix Table 3. Sample characteristics (N = 4,508) before and after multiple imputation.** | | |
| --- | --- | --- |
|  | **Without imputation** | **With imputation** |
| **Age (years), mean (SD)** | 27.0 (5.6) | 27.0 (5.6) |
| **Self-reported maternal race and ethnicity** |  |  |
| **Non-Hispanic Asian** | 62.1% | 62.1% |
| **Non-Hispanic Black** | 13.8% | 13.8% |
| **Hispanic** | 16.3% | 16.3% |
| **Non-Hispanic White** | 3.0% | 3.0% |
| **Other** | 4.7% | 4.7% |
| **Educational attainment** |  |  |
| **High school or less** | 18.6% | 18.6% |
| **Some college or associate/tech** | 31.0% | 30.9% |
| **College or above** | 50.4% | 50.4% |
| **Health insurance** |  |  |
| **Private insurance** | 54.6% | 54.6% |
| **Public insurance** | 27.7% | 27.7% |
| **Self-pay or other source** | 17.7% | 17.7% |
| **Area deprivation index 2015, mean (SD)** | 47.6 (30.4) | 48.0 (30.4) |
| **Body mass index (kg/m2), mean (SD)** | 26.6 (6.5) | 26.6 (6.5) |
| **Systolic blood pressure (mmHg), mean (SD)** | 109.5 (10.9) | 109.5 (10.9) |
| **Diastolic blood pressure (mmHg), mean (SD)** | 67.3 (8.4) | 67.3 (8.4) |
| **Glucose (mg/dL), mean (SD)** | 88.1 (16.1) | 88.1 (16.1) |
| **Non-HDL cholesterol (mg/dL), mean (SD)** | 72.4 (15.3) | 72.4 (15.2) |
| **Sleep duration (hours per night), mean (SD)** | 7.9 (1.3) | 8.0 (1.3) |
| **Healthy Eating Index-2010, mean (SD)** | 62.9 (12.7) | 62.4 (12.8) |
| **Physical activity (minutes per week), mean (SD)** | 186.9 (174.3) | 183.4 (166.1) |
| **Tobacco Use** |  |  |
| **Current user** | 16.1% | 16.0% |
| **Prior user** | 23.1% | 23.1% |
| **Ever used** | 60.8% | 60.9% |

| **Appendix Table 4. Characteristics by ADI tertiles.** | | | | |
| --- | --- | --- | --- | --- |
|  | **Tertile 1**  **(low deprivation)** | **Tertile 2** | **Tertile 3**  **(high deprivation)** | **p-value** |
| **Area Deprivation Index 2015**  **Mean (SD)**  **Median (IQR)** | 15.7 (8.0)  16 (10-23) | 44.3 (9.6)  44 (36-52) | 85.4 (11.6)  89 (75-96) | <0.001  <0.001 |
| **Age (years), mean (SD)** | 29.5 (5.0) | 27.3 (5.2) | 24.2 (5.3) | <0.001 |
| **Self-reported maternal race and ethnicity** |  |  |  | <0.001 |
| **Non-Hispanic Asian** | 75.3% | 74.7% | 36.2% |  |
| **Non-Hispanic Black** | 3.3% | 7.5% | 31.0% |  |
| **Hispanic** | 12.5% | 11.1% | 25.4% |  |
| **Non-Hispanic White** | 5.5% | 2.0% | 1.3% |  |
| **Other** | 3.3% | 4.8% | 6.1% |  |
| **Educational attainment** |  |  |  | <0.001 |
| **High school or less** | 5.5% | 13.9% | 36.9% |  |
| **Some college or associate degree** | 19.8% | 35.4% | 38.2% |  |
| **College or above** | 74.7% | 50.8% | 24.9% |  |
| **Health insurance** |  |  |  | <0.001 |
| **Private insurance** | 62.3% | 65.2% | 36.0% |  |
| **Public insurance** | 11.4% | 18.2% | 53.9% |  |
| **Self-pay or other source** | 26.3% | 16.5% | 10.0% |  |

| **Appendix Table 5. Associations between Area Deprivation Index (ADI) and overall and component Life’s Essential 8 scores in early pregnancy without data imputation.** | | | | | | | | | |
| --- | --- | --- | --- | --- | --- | --- | --- | --- | --- |
|  | **Diet** | **Physical activity** | **Tobacco use** | **Sleep health** | **BMI** | **Serum lipid level** | **Serum glucose level** | **Blood pressure** | **LE8** |
|  | Percentile score on the HEI-2010 | Minutes per week | Smoking status | Hours per night | kg/m^2^ | mg/dL | mg/dL | Systolic/diastolic, mmHg | **%** |
| N | 7955 | 6752 | 9571 | 7406 | 9421 | 4202 | 4334 | 9405 | 2134 |
| **Adjusted Least Square Means**  **(95% CI)^1^** |  |  |  |  |  |  |  |  |  |
| **ADI tertile** |  |  |  |  |  |  |  |  |  |
| Tertile 1 (low deprivation) | 74.05  (71.58, 76.51) | 74.11  (69.17, 79.05) | 70.32  (63.18, 77.45) | 89.30  (87.93, 90.66) | 80.66  (78.16, 83.16) | 86.92  (84.30, 89.54) | 88.39  (85.18, 91.60) | 92.70  (90.74, 94.65) | 84.68  (83.63, 85.72) |
| Tertile 2 | 71.55  (67.21, 75.89) | 70.40  (66.20, 74.59) | 72.01  (63.65, 80.38) | 89.10  (87.78, 90.42) | 73.78  (71.79, 75.76) | 87.14  (85.14, 89.14) | 87.53  (85.15, 89.91) | 89.96  (88.10, 91.83) | 81.75  (79.93, 83.57) |
| Tertile 3 (high deprivation) | 68.23  (63.70, 72.77) | 71.90  (69.75, 74.05) | 68.59  (58.70, 78.48) | 87.66  (86.62, 88.70) | 70.16  (64.37, 75.95) | 86.23  (83.29, 89.16) | 85.30  (82.23, 88.36) | 89.89  (87.57, 92.20) | 79.15  (76.66, 81.65) |
| **Adjusted Beta coefficient**  **(95% CI)^2^** |  |  |  |  |  |  |  |  |  |
| **ADI tertile** |  |  |  |  |  |  |  |  |  |
| Tertile 1 (low deprivation) | Ref. | Ref. | Ref. | Ref. | Ref. | Ref. | Ref. | Ref. | Ref. |
| Tertile 2 | -2.50*  (-4.81, -0.18) | -3.71  (-7.64, 0.22) | 1.70  (-2.67, 6.07) | -0.19  (-1.98, 1.60) | -6.88*  (-8.83, -4.93) | 0.22  (-3.10, 3.53) | -0.86  (-3.59, 1.87) | -2.73*  (-3.69, -1.77) | -2.92*  (-4.19, -1.66) |
| Tertile 3 (high deprivation) | -5.81*  (-9.80, -1.83) | -2.21  (-6.52, 2.11) | -1.73  (-9.71, 6.26) | -1.64  (-3.56, 0.28) | -10.50*  (-17.32, -3.67) | -0.69  (-4.37, 2.98) | -3.09  (-7.05, 0.86) | -2.81*  (-4.94, -0.68) | -5.52*  (-7.92, -3.12) |
| ^1^Means were adjusted for maternal age (continuous), self-reported race and ethnicity as a social determinant (White, Black, Hispanic, Asian, other), and educational attainment.  ^2^Models was adjusted for maternal age (continuous), self-reported race and ethnicity as a social determinant (White, Black, Hispanic, Asian, other), and educational attainment.  *p<0.05.  Abbreviations: BMI (body mass index) | | | | | | | | | |
